# The complex analysis of X-ray mesh scans for macromolecular crystallography

**DOI:** 10.1107/S2059798318002735

**Published:** 2018-04-06

**Authors:** Igor Melnikov, Olof Svensson, Gleb Bourenkov, Gordon Leonard, Alexander Popov

**Affiliations:** a European Synchrotron Radiation Facility, BP 220, 38043 Grenoble, France; b European Molecular Biology Laboratory, Hamburg Outstation, Notkestrasse 85, 22607 Hamburg, Germany

**Keywords:** macromolecular crystallography, X-ray mesh scans, sample localization, data-collection strategies, *MeshBest*

## Abstract

In macromolecular crystallography, mesh (raster) scans are carried out either as part of X-ray-based crystal-centring routines or to identify positions on the sample holder from which diffraction images can be collected. Here, the methods used in *MeshBest*, software which automatically analyses diffraction images collected during a mesh scan and produces a two-dimensional crystal map showing estimates of the dimensions, centre positions and diffraction qualities of each crystal contained in the mesh area, are presented. Sample regions producing diffraction images resulting from the superposition of more than one crystal are also distinguished from regions with single-crystal diffraction. The applicability of the method is demonstrated using several cases.

## Abstract 2

A method and software program, *MeshBest*, for the detection of individual crystals based on two-dimensional X-ray mesh scans are presented.

## 1. Introduction   

In X-ray crystallography, samples vary in size, in shape and in diffraction strength. The experiments carried out for data collection in macromolecular crystallography (MX) can be optimized (Bourenkov & Popov, 2006 ▸) *via* pre-interrogation of the sample(s). Such pre-interrogation should provide essential information concerning the shape, size, position and diffraction strength of the crystal.

The conventional way of localizing crystals in a sample holder and estimating crystal size is to use optical microscopy. However, when dealing with large numbers of ever smaller crystals, often immersed in a nonhomogeneous strongly refracting medium, experimentalists are confronted with the limitations of the classical approach. A number of advanced optical schemes have therefore been proposed, including fluorescence microscopy (Vernede *et al.*, 2006 ▸; Madden *et al.*, 2011 ▸; Newman *et al.*, 2016 ▸), second-harmonic generation (Haupert & Simpson, 2011 ▸; Kissick *et al.*, 2013 ▸; Madden *et al.*, 2011 ▸, 2013 ▸; Newman *et al.*, 2016 ▸) and Raman spectroscopy (von Stetten *et al.*, 2015 ▸). Alternatively, the use of techniques such as full-field (Brockhauser *et al.*, 2008 ▸; Warren *et al.*, 2013 ▸) or scanning transmission (Wojdyla *et al.*, 2016 ▸) X-ray microscopy have also been reported.

However, thanks to recent technological breakthroughs in the diffractometers available at synchrotron MX beamlines (Mueller-Dieckmann *et al.*, 2015 ▸; Allan *et al.*, 2015 ▸; Owen *et al.*, 2016 ▸) and the development of pixel-array detectors (Henrich *et al.*, 2009 ▸) capable of fast data-acquisition rates in a shutterless fashion (Hülsen *et al.*, 2006 ▸), by far the most popular approach for crystal detection in MX is the two-dimensional mesh (raster) X-ray diffraction scan, which has been implemented with slight variations on several MX synchrotron beamlines worldwide (Cherezov *et al.*, 2009 ▸; Bowler *et al.*, 2010 ▸; Aishima *et al.*, 2010 ▸; Svensson *et al.*, 2015 ▸; Zander *et al.*, 2015 ▸; Wojdyla *et al.*, 2016 ▸; Gati *et al.*, 2014 ▸).

During such a mesh scan, the sample holder is translated under the exposure of the X-ray beam and diffraction images are accumulated at each point of a pre-defined two-dimensional grid, the number of points in which is defined by the size of the incident X-ray beam and the dimensions of the area to be scanned. The resulting diffraction images are then analysed [*i.e.* using *DISTL* and *Spotfinder* (Zhang *et al.*, 2006 ▸), *CrystFEL* (White *et al.*, 2016 ▸), *MOSFLM* (Battye *et al.*, 2011 ▸), *XDS* (Kabsch, 2010 ▸), *EDNA*/*BEST* (Bourenkov & Popov, 2010 ▸) *etc.*] for the presence of diffraction spots and the resolution range to which they extend. On the ESRF MX beamlines the image analysis is carried out by the program *Dozor* (Svensson *et al.*, 2015 ▸; Zander *et al.*, 2015 ▸), which estimates the diffraction signal based on empirical radial scattering patterns of protein crystals (Popov & Bourenkov, 2003 ▸). The diffraction signal in each particular image is then ranked and the results are displayed as a two-dimensional heat map.

The major advantage of an X-ray mesh scan is that the diffraction images directly show well diffracting regions in the sample holder on the heat map. However, when many crystals are present the heat map does not provide information on which adjacent regions in the map, if any, belong to a given individual crystal (*i.e.* the heat map does not provide specific information concerning the number, size and disposition of crystals contained in a sample holder). Such information can be extremely important in planning MX experiments, and here a method is presented for the recognition, based on the X-ray mesh-scan technique and image analysis carried out by *Dozor*, of protein crystals contained in the sample area. The method developed both distinguishes regions belonging to different crystals and detects regions where several crystals are exposed to the beam simultaneously, producing multi-crystal diffraction patterns. The implemented in the program *MeshBest*, which outputs descriptions of all individual crystals in the sample area, including crystal sizes, shapes, centre positions and diffraction strengths.

## 2. Methods   

**Figure 1:**
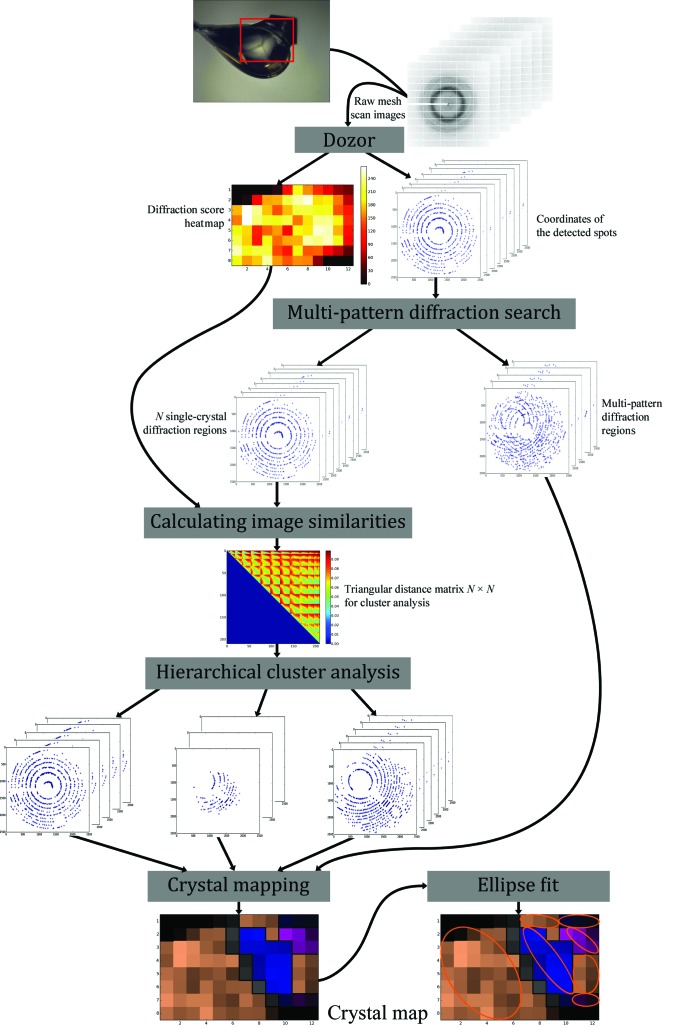
Overview of the workflow of the method. Each X-ray mesh scan produces *N*_rows_ × *N*_columns_ diffraction images. These are individually analysed by *Dozor*, which produces an estimate of the diffraction signal and determines a list of diffraction-spot coordinates and their partial intensities in each image. *MeshBest* then carries out the analyses described in the main text.

The *MeshBest* workflow is presented in Fig. 1 ▸ and comprises three major steps. Firstly, any mesh-scan images that contain multi-crystal diffraction patterns are detected. This is performed by analysing diffraction vector statistics for each diffraction image (§[Sec sec2.1]2.1). Secondly, diffraction images containing diffraction from only one crystal are analysed and those belonging to the same crystal are grouped (§[Sec sec2.2]2.2). Finally, the sizes and dispositions of all single crystals contained in the sample loop are described using an elliptical shape approximation (§2.3). Note that rotation of the sample during a mesh scan is not preferred; acquiring still images would simplify the analysis. However, the mesh scans presented here were acquired with slight rotation, which was caused by the need to trigger detector readout on some beamlines.

### 2.1. Detecting superposition of multiple diffraction patterns   

**Figure 2:**
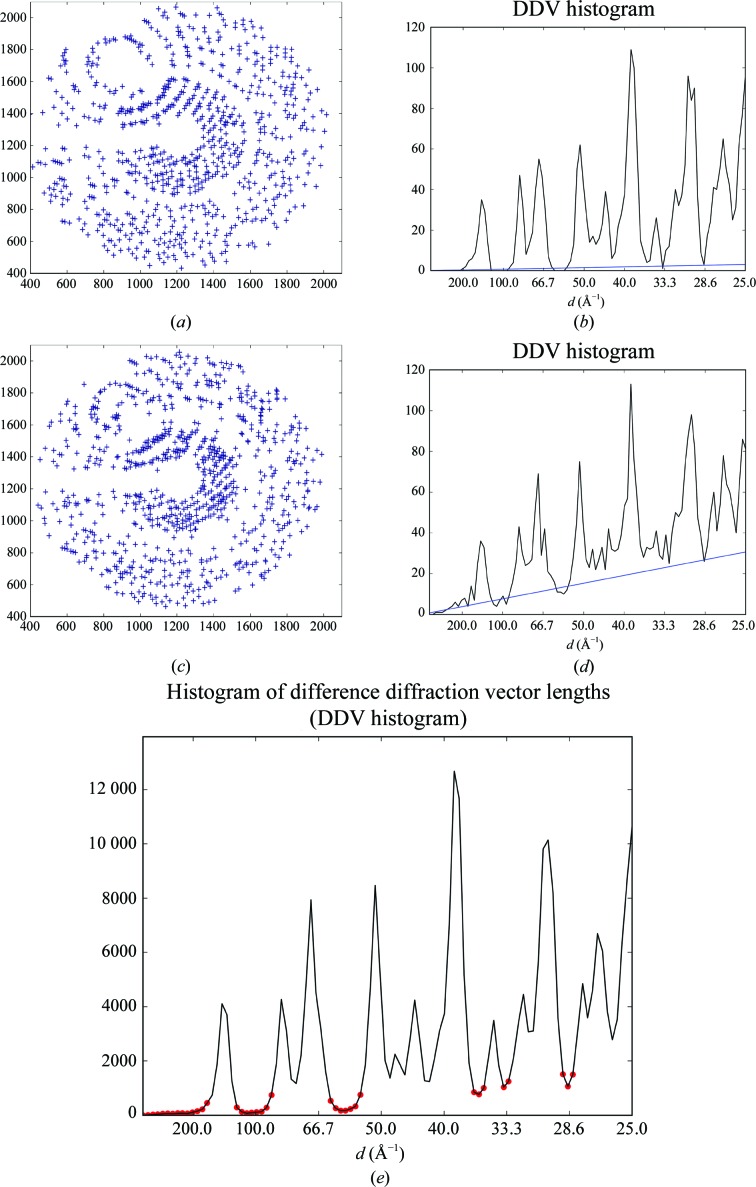
Multi-pattern diffraction analysis. (*a*, *b*) A spot diagram in the detector plane for an example of single-pattern diffraction (*a*) and the corresponding DDV histogram (*b*) for a thermolysin crystal (§[Sec sec3.2]3.2). (*c*, *d*) A spot diagram in the detector plane for an example of multi-pattern diffraction (*c*) and the corresponding DDV histogram (*d*) for a thermolysin crystal (§[Sec sec3.2]3.2). Blue lines in the histograms show the fitted baselines. (*e*) A cumulative DDV histogram of all images of the mesh scan for a thermolysin crystal (§[Sec sec3.2]3.2), with the determined baseline regions depicted by red circles. The numbers in the histograms are presented for 100 bins in the interval of the analysis.

In the case of diffraction from a single crystal, the lengths of the difference diffraction vectors (DDVs; the vectors between nodes of the reciprocal lattice of a crystal) observed in a diffraction pattern can only have certain values because all of the diffraction spots are at the positions of the nodes of the reciprocal lattice of the crystal. The distribution of DDV lengths thus consists of several peaks, with each peak representing a particular subset of distances between reciprocal-lattice nodes (Figs. 2 ▸ *a* and 2 ▸ *b*). The frequency at which these peaks occur depends on the crystal orientation in the X-ray beam. The superposition of two or more diffraction patterns in a single diffraction image leads to the appearance of additional DDVs between the different lattices. The distribution of lengths of inter-lattice DDVs appears to be approximately linear in the relatively short range of distances analysed here (1–40 × 10^−3^ Å^−1^). The resulting distribution will comprise the peaks for intra-lattice distances imposed on a monotonous, roughly linear baseline (Figs. 2 ▸ *c* and 2 ▸ *d*). As the slope of the baseline is proportional to the total number of DDVs between different lattices in a diffraction image, fitting the baseline (*i.e.* determining its slope, *k*_0_) thus allows a quantitative judgement as to whether a diffraction image contains multi-pattern diffraction. However, the more spots that are present in the image and the more tightly they are packed, the more DDVs will be counted. To account for this, we use the following to quantitatively assess multi-pattern diffraction,
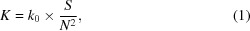
which is the slope of the baseline normalized by the spot density *N*/*S* and the total number of spots *N*. Here, *k*_0_ is the slope value of the baseline fit (expressed in Å), *S* is the area of the Ewald sphere cap containing all of the spots (expressed in Å^−2^) and *N* is the number of detected spots in the image. *K* is therefore expressed in Å^−1^.

It can be shown (see the Supporting Information) that *K* increases with the increasing contribution of satellite crystal spots to a diffraction image. It can also be shown (Supporting Information) that a multi-crystal diffraction pattern can be successfully indexed when *K* < 1.4 × 10^−4^ Å^−1^, corresponding to the case where satellite crystals contribute three times fewer spots than the main crystal. In *MeshBest*, images with *K* > 1.4 × 10^−4^ Å^−1^ are thus marked as originating from multi-crystal diffraction.

To determine the value of *K* for a given diffraction image, *MeshBest* constructs a histogram of difference vector lengths (a DDV histogram). The diffraction vectors are calculated from the spot coordinates in the image. DDV lengths are then calculated and a 100-bin histogram is computed for every diffraction image in the boundaries from 0 to (25)^−1^ Å^−1^, and the baseline is estimated by a linear fit in the baseline regions (Figs. 2 ▸ *b* and 2 ▸ *d*). Baseline regions (red circles in Fig. 2 ▸ *e*) are selected as local minima in the sum of all histograms of the mesh using the algorithm for automatic multiscale-based peak detection (AMPD; Scholkmann *et al.*, 2012 ▸).

### 2.2. Discriminating between distinct crystals   

Once *MeshBest* has identified and discarded all images with unacceptable multi-pattern diffraction, the program searches for the remaining regions of the mesh scan that produce diffraction signal and which are geometrically interconnected. Every pair of images in each region is then analysed to determine how similar their diffraction patterns are.

This is performed by comparing the positions of common diffraction spots in the two images. In order to be independent of instrument parameters, the angle between the scattered rays corresponding to the diffraction spots in the images is taken as the criterion for spot-position similarity. The image with the smaller number of spots is taken as the reference image. We define the distance score *D* (2) between two images as the root-mean-square value of the angular deviations between the scattered rays corresponding to spot positions,
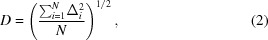
where *N* is the number of spots in the reference image and Δ*_i_* is the angle expressed in degrees between the scattered rays passing through the centres of the *i*th spot in the reference image and the corresponding spot in the compared image.

For every diffraction spot in the reference image, the corresponding spot in the second (compared) image is searched for. Obviously, the centre positions of the same diffraction spot in two mesh-scan images resulting from the same crystal orientation should be relatively close. In *Dozor*, diffraction spots are integrated over a square of 3 × 3 detector pixels by default. Guided by this consideration, in *MeshBest* it is adopted independently of the instrumentation that spots in two images are not the same if their diffracted rays differ more than 0.1° in orientation. If any spot in the compared image is found within 0.1° of the reference spot Δ*_i_* is calculated for the two spots, otherwise Δ*_i_* is set to 0.1°. Finally, the distance score (2), which is limited to 0.1° and is independent of the numbers of spots in the two images being compared, is calculated.

**Figure 3:**
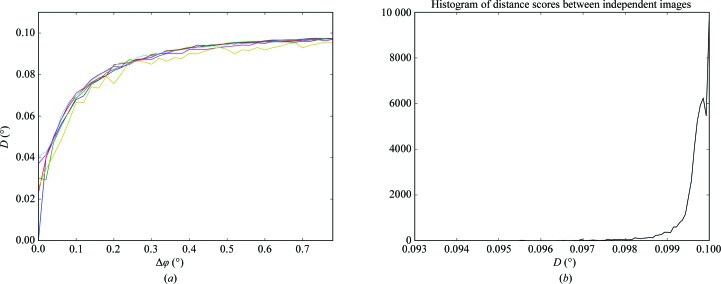
(*a*) The behaviour of the distance score *D* as a function of the crystal rotation between frames (Δφ). In this experiment several standard 50-frame data collections were carried out from the same crystal of thaumatin using the same data-collection parameters except for the beam transmission, which was changed to imitate different diffraction strengths. *D* was then calculated between pairs of images and plotted against Δφ. The rotation per frame was 0.02°. The average crystal mosaicity was 0.04° as determined by *XDS* (Kabsch, 2010 ▸). Different colours show the results with different X-ray beam transmissions (from 100% in blue to 0.1% in yellow). (*b*) A histogram of the distance scores obtained between randomly selected distant images from the same rotational data set. Several data sets from thaumatin, thermolysin and lysozyme crystals were used with different crystal mosaicities and their histograms were summed.

Fig. 3 ▸(*a*) shows *D* as a function of the angular difference between crystal orientations when pairs of images from a standard rotation data collection are compared. *D* tends to a value of 0.1° as the compared images are taken at more and more distant orientations of the crystal in the X-ray beam. The random error in the determination of the distance score can be estimated from the histogram of the distance scores between non-adjacent images in the rotational data set, as shown in Fig. 3 ▸(*b*). The histogram was calculated based on comparing distant images of the same rotational data set and was averaged on different protein crystals, and shows only a small deviation from the expected value of *D* = 0.1° which is owing to random spot-position coincidence. In *MeshBest*, a criterion *D* > 0.093° is introduced to distinguish diffraction images arising from different crystals. The value of the threshold is chosen as ten standard deviations from the centre of the peak in the histogram of the distance scores between independent images (Fig. 3 ▸ *b*).

In each interconnected region, the values of *D* (equation 2, see below) for every pair of images are then accumulated in an upper triangular matrix. Hierarchical cluster analysis (HCA; Sneath & Sokal, 1962 ▸) is then performed on the matrix using the ‘average’ linkage method, which takes the average distance between the elements of two different clusters as the distance between them. If a mesh scan is measured with rotation, then the linkage method is slightly modified, applying zero weights to pairs of images taken at crystal orientations that differ by more than 0.5° when calculating the ‘average’ linkage (see, for example, §[Sec sec3.2]3.2). The final clusters are formed by applying a cluster distance cutoff of 0.093° to the dendrogram. Subsequently, those images that have been clustered together are treated as stemming from individual crystals.

### 2.3. Creating a crystal map and fitting elliptical crystal shapes   

**Figure 4:**
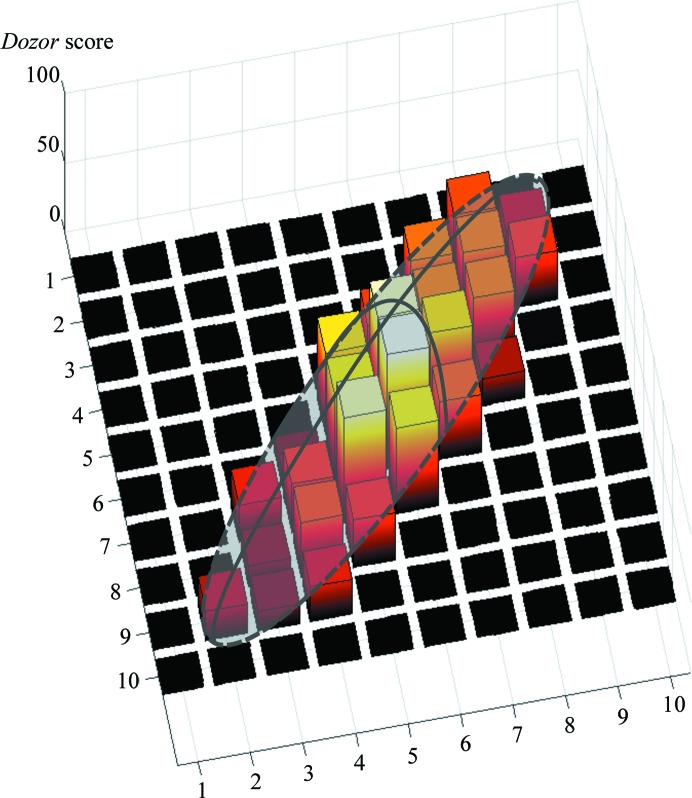
A schematic view of a semi-ellipsoid shape fitted to the three-dimensional representation of the heat map, with the *Dozor* score indicated on the third axis.

Once the HCA step has been carried out, *MeshBest* estimates the size and shape of each individual crystal region determined (Fig. 4 ▸). This is an essential step for the determination of optimal data-collection strategies. For this, two-dimensional projections of the individual crystals identified by HCA (§[Sec sec2.2]2.2) are approximated by an elliptical shape. However, as different regions of a crystal may have different diffraction signals, additional information concerning this should be taken into account when describing their two-dimensional areas. For this reason, in crystal maps produced by *MeshBest* the *Dozor* scores for each grid point are plotted on a third axis perpendicular to the mesh-scan plane, and the resulting three-dimensional diagram of each crystal is approximated using a semi-ellipsoid shape (see Fig. 4 ▸) parameterized by five quantities. The optimization algorithm (Storn & Price, 1997 ▸) was chosen based on several test cases and is used to fit the shape into the diagram region by minimization of the function
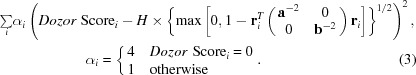
where α_*i*_ is a weight coefficient penalizing the presence of nonzero fit on the mesh-scan areas with no diffraction,
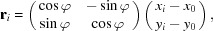
where *x_i_*, *y_i_* are the mesh-scan coordinates of the *i*th image, and *H*, *x*_0_, *y*_0_, *a*, *b* and φ are the parameters of the ellipsoid being optimized.

In this way, each crystal region is approximated by an ellipsoid where *H* represents the average diffraction strength. The individual crystals identified are sorted in a list according to the integral diffraction signal *I* = (2/3)π*abH*. This list can then be used to select the best diffracting crystals for subsequent data collection. To visualize the result, *MeshBest* creates a crystal map in which crystal regions are labelled in one of nine colours tinted according to the diffraction signal (see, for example, Fig. 8*f*). The use of a standard palette of nine colours allows neighbouring regions of different crystals to be clearly separated by eye. The appropriate ellipses are drawn over each individual crystal region. Those regions of the mesh scan that are identified as producing unacceptable multi-pattern diffraction images are coloured grey.

## 3. Test results   

The program *MeshBest* was implemented as a Python module using standard (NumPy, SciPy) libraries. In order to demonstrate the applicability of *MeshBest* and its performance, we present four different experiments here. These include *MeshBest* analyses of a large, homogeneous crystal (§[Sec sec3.1]3.1) and of a disordered crystal with satellites (§[Sec sec3.2]3.2), the analysis of a mesh scan performed prior to subsequent multi-crystal data collection on a sample holder containing membrane-protein crystals buried in opaque lipidic mesophase (§[Sec sec3.3]3.3) and a mesh scan from a sample holder containing a crystal mess with ‘dirty’ diffraction patterns (§[Sec sec3.4]3.4).

Table 1 ▸ shows the data-collection parameters used in these experiments. Data were collected on ESRF beamlines ID23-1, ID29 and MASSIF-3 (Nurizzo *et al.*, 2006 ▸; de Sanctis *et al.*, 2012 ▸; Theveneau *et al.*, 2013 ▸) using X-ray beams with near-Gaussian profiles and a beam size ranging from 10 to 50 µm FWHM. Prior to data collection, grid locations and sizes were defined manually in the *MxCuBE*2 beamline-control interface (Gabadinho *et al.*, 2010 ▸; de Sanctis *et al.*, 2016 ▸), with the number of grid points in each direction automatically calculated based on the incident X-ray beam size. Mesh scans were then carried out in a shutterless fashion and diffraction images were collected using PILATUS 6M or PILATUS3 2M pixel-array detectors (Dectris, Baden, Switzerland). In all experiments there was a constraint, defined by the need to trigger detector readout, to perform a slight rotation around the ω (goniometer rotation) axis when collecting each row of the mesh.

### 3.1. A large, homogeneous crystal   

**Figure 5:**
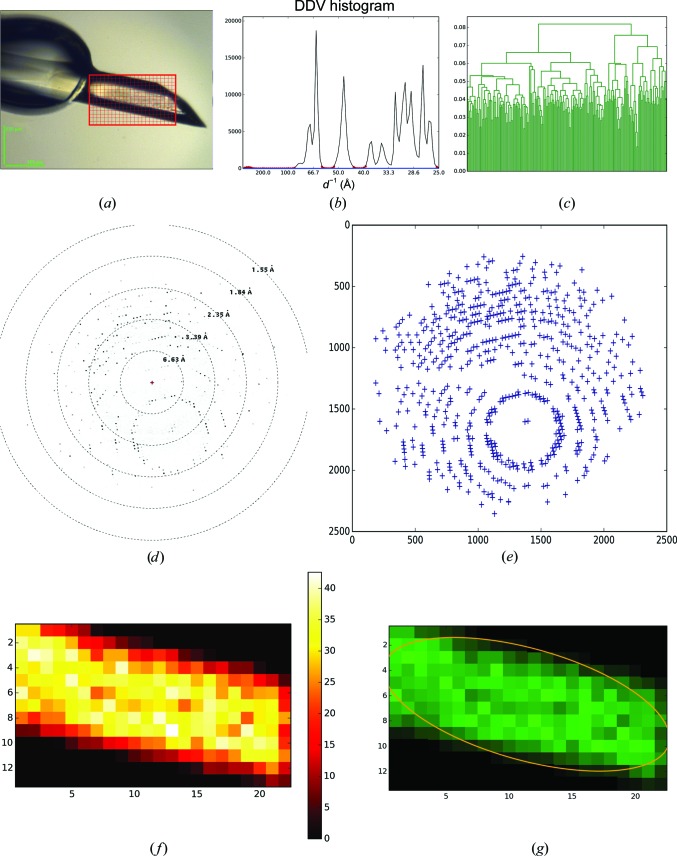
*MeshBest* analysis of the mesh scan of a large, homogeneous, nearly perfect crystal of trypsin. (*a*) A snaphot of the crystal as mounted on the beamline goniometer; the red rectangular mesh indicates the area and the dimensions of the mesh scan. (*b*) Cumulative DDV histogram used in multi-pattern diffraction analysis, with the baseline regions determined marked with red circles. The blue line shows the fitted baseline of the cumulative histogram. Its slope indicates the absence of multi-pattern diffraction in the images of the mesh scan. (*c*) Dendrogram based on HCA of mesh-scan images (see §2.2); the colours correspond to the crystal map in (*g*). (*d*) A sample of a raw diffraction image from the mesh scan. (*e*) A diagram of spots detected by *Dozor* corresponding to the image in (*d*). (*f*) *Dozor*-score heat map of the mesh scan. (*g*) A crystal map of the mesh scan generated by *MeshBest*.

A large crystal of bovine trypsin (Sigma–Aldrich) was cryocooled and mounted on the beamline. The mesh scan was performed in the area shown in Fig. 5 ▸(*a*). No signs of multi-crystal diffraction were recognized in the images of this mesh scan (Fig. 5 ▸ *b*). Based on the 210 interconnected diffracting regions of the mesh scan, a distance matrix 210 × 210 in size was calculated. As might be expected based on the appearance of the crystal in the region of the mesh scan, subsequent HCA (Fig. 5 ▸ *c*) determined this to be a single crystal and an ellipse was fitted to the crystal shape (Fig. 5 ▸ *g*).

### 3.2. A large disordered crystal with satellites   

**Figure 6:**
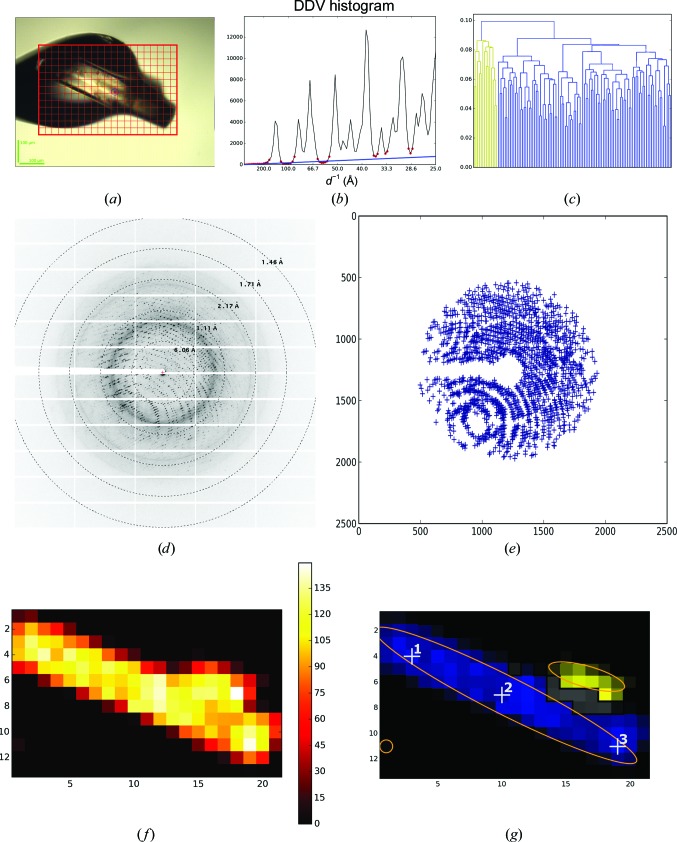
*MeshBest* analysis of the mesh scan of a large thermolysin crystal. (*a*) A snapshot of the sample as mounted on the beamline goniometer; the red rectangular mesh indicates the area and the dimensions of the mesh scan. (*b*) Cumulative DDV histogram used in multi-pattern diffraction analysis, with determined baseline regions marked with red circles. The blue line shows the fitted baseline of the cumulative histogram, the slope of which indicates the presence of multi-pattern diffraction in the images of the mesh scan. (*c*) Dendrogram based on HCA of mesh-scan images (see §[Sec sec2.2]2.2); the colours correspond to the crystal map in (*g*). (*d*) A sample of a raw diffraction image from the region of the mesh scan with multi-pattern diffraction [shown in grey in (*g*)]. (*e*) A diagram of spots detected by *Dozor* corresponding to the image in (*d*). (*f*) *Dozor*-score heat map of the mesh scan. (*g*) A crystal map of the mesh scan generated by *MeshBest*. Numbered white crosses show the positions at which the partial data sets were collected to determine lattice orientations.

A large crystal of thermolysin (from *Bacillus thermoproteo­lyticus*; Sigma–Aldrich) was analysed with a mesh scan as shown in Fig. 6 ▸. The *Dozor*-score map (Fig. 6 ▸ *f*) contained one large connected area characterized by strong diffraction signal plus a weak diffraction image apparently originating from a tiny satellite crystal (bottom left corner in Fig. 6 ▸ *f*). Superposition of the diffraction patterns, marked in grey in Fig. 6 ▸(*g*), was detected within a small area. The remaining part of the large area, comprising 115 mesh points at which single-crystal diffraction was detected, was interconnected (although the connectivity is difficult to notice in Fig. 6 ▸ *g*). Subsequent HCA on the 115 × 115 similarity matrix (Fig. 6 ▸ *c*) divided the area into two clusters, which are marked in blue and yellow in Fig. 6 ▸(*g*). In accordance with a microphotograph (Fig. 6 ▸ *a*), the case is interpreted well, as proven by the presence of a satellite crystal (yellow) on top of the major rod-shaped single crystal (blue). The projections of the two crystals onto the mesh plane are superimposed in the grey area. Note that the analysis was carried out by *MeshBest* in a fully automatic manner.

**Figure 7:**
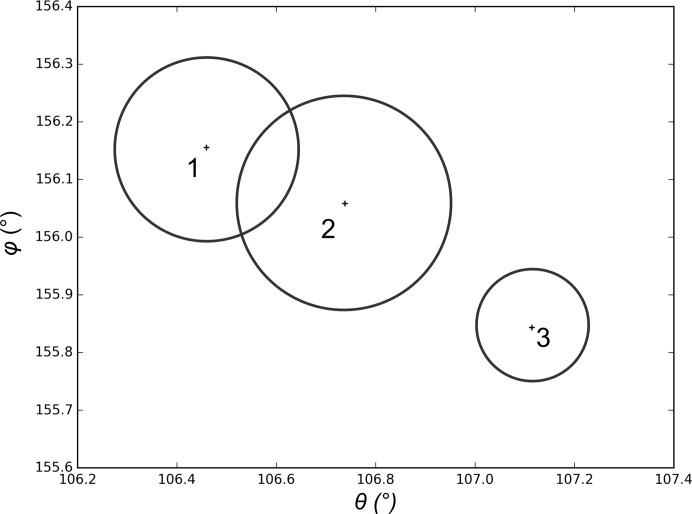
A diagram showing the orientations of the c* vector of the reciprocal lattice in different regions of the thermolysin crystal shown in Fig. 6 ▸. The orientations are presented here by two Euler angles, with the values calculated from the data collected at the positions given by the corresponding numbers in Fig. 6 ▸(*g*). The circles denote an error estimate of the c* direction: the radii are equal to the standard deviations of the spindle position as reported by *XDS*.

A close inspection of the dendrogram (Fig. 6 ▸ *c*) suggests that by choosing a linkage threshold of <0.8 the major cluster could, in fact, be split into three or four smaller clusters (*i.e.* the large crystal is not completely homogeneous). One might well expect such behaviour in a crystal spanning across a cryoloop and extending away from the cryo-solution matrix, and in order to verify the automatic *MeshBest* interpretation we carried out additional data collections after centring at the three points indicated in Fig. 6 ▸(*g*). 50 images of 0.1° rotation data were collected at each position and the crystal orientation in the X-ray beam was determined using *XDS* (Kabsch, 2010 ▸). Fig. 7 ▸ shows the orientations of the c* vector, expressed in Euler angles, at the different crystal positions tested. Clearly, the crystal has a discrepancy in lattice orientation between the two ends of about 1°. For comparison, the misorientation between the extreme ends of the trypsin crystal in Fig. 5 ▸ was 0.06° as determined using *XDS* on the images of the mesh scan; this suggests that the large thermolysin crystal should be annotated as being more than one sample. However, as the misorientation shown in Fig. 7 ▸ occurs gradually over the length of the crystal *MeshBest* defines it as being a single crystal, as almost certainly would a human experimenter.

### 3.3. Multi-crystal data collection in the *MeshAndCollect* pipeline   

**Figure 8:**
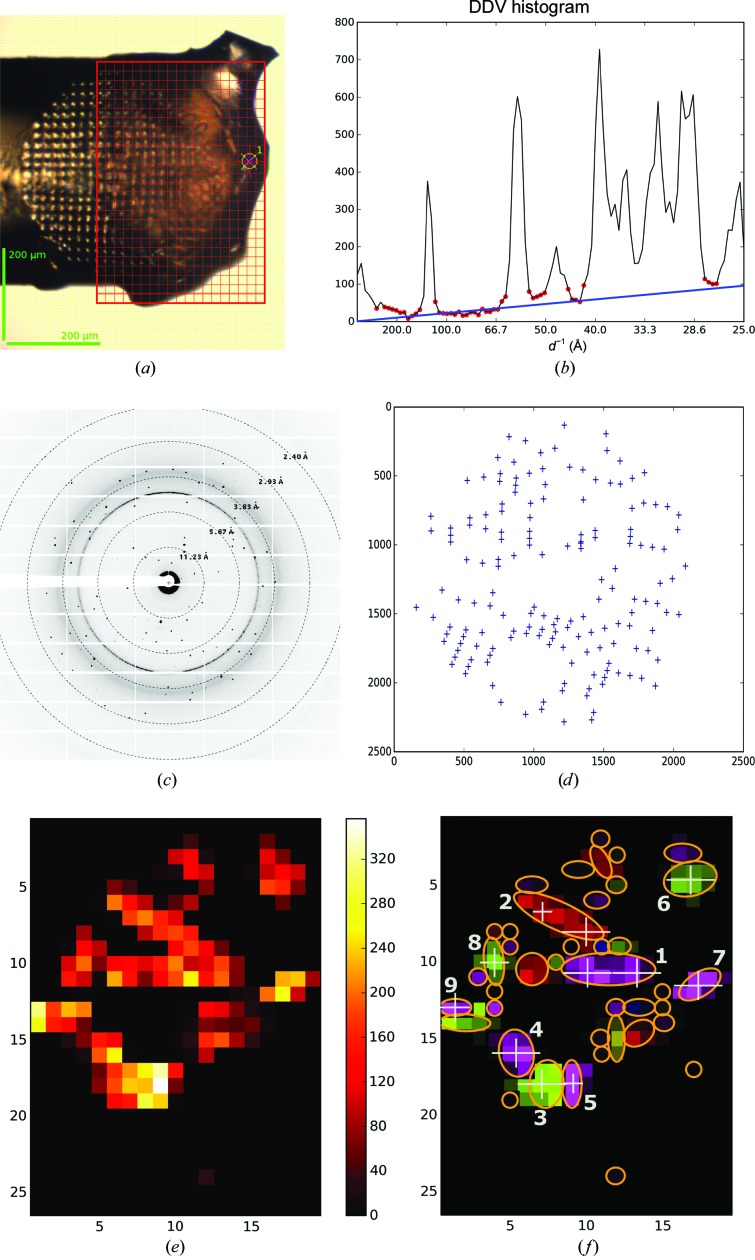
*MeshBest* analysis of the mesh scan of NarQ crystals for subsequent multi-crystal data collection. (*a*) A snapshot of the sample as mounted on the beamline goniometer; the red rectangular mesh indicates the area and the dimensions of the mesh scan. (*b*) Cumulative DDV histogram used in multi-pattern diffraction analysis, with baseline regions marked with red circles. The blue line shows the fitted baseline of the cumulative histogram, the slope of which indicates the presence of multi-pattern diffraction in the images of the mesh scan. (*c*) An example of a raw diffraction image from the mesh scan. (*d*) A diagram of spots detected by *Dozor* corresponding to the image in (*d*). (*e*) *Dozor*-score heat map of the mesh scan. (*f*) A crystal map of the mesh scan generated by *MeshBest*. The crystals used for data collection are numbered according to their diffraction-score rank. White crosses with the size of the beam aperture mark the positions at which partial data sets were collected.

Crystals of a recombinant transmembrane construct of the nitrate/nitrite-dependent histidine kinase NarQ from *Escherichia coli* (R50K mutant; Gushchin *et al.*, 2017 ▸) were harvested in a micromesh sample holder (Fig. 8 ▸ *a*) and a mesh scan was performed. The mesh-scan area consisted of multiple connected diffracting regions (see Fig. 8 ▸ *e*). HCA divided these areas into 41 individual crystals, the dimensions and the centre coordinates of which were determined using elliptical shape approximation (§[Sec sec2.3]2.3; shown in different colours in Fig. 8 ▸ *f*). Subsequently, 20° of rotation data (φ in the range from −10 to 10°) were collected from the best crystals as ranked by the integral diffraction score given by *MeshBest*. Each data set was collected using a beam size (white crosses in Fig. 8 ▸ *f*) chosen in accordance with the dimensions of crystal size approximations. Crystals 1 and 2 were significantly larger than a particular beam size, and here two data collections were performed at different positions within each of the two crystals. A total of 11 partial data sets were acquired. The diffraction images from each partial data set were then processed with *XDS* and *XSCALE* (Kabsch, 2010 ▸), with HCA (Giordano *et al.*, 2012 ▸) suggesting the rejection of one partial data set that correlated poorly with the others. The final data set produced (Table 2 ▸) extends to a resolution of 2.0 Å.

### 3.4. The case of a crystal mishmash   

**Figure 9:**
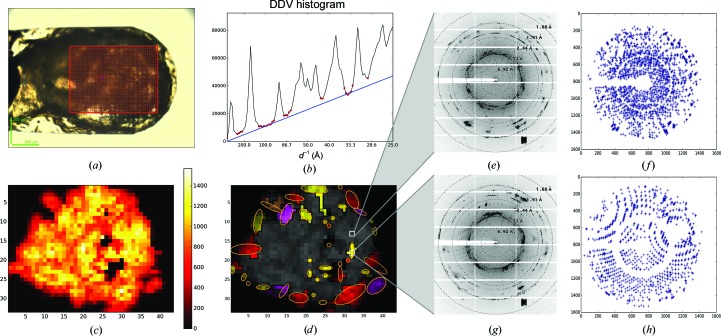
*MeshBest* analysis of the mesh scan of thaumatin crystals harvested in a way which produces a crystal ‘mismash’. (*a*) A snapshot of the sample holder as mounted on the beamline goniometer; the red rectangle indicates the area of the mesh scan. (*b*) Cumulative DDV histogram used in multi-pattern diffraction analysis, with determined baseline regions marked with red circles. The blue line shows the fitted baseline of the cumulative histogram, the slope of which indicates the presence of multi-pattern diffraction in the images of the mesh scan. (*c*) *Dozor*-score heat map of the mesh scan. (*d*) A crystal map of the mesh scan generated by *MeshBest*. Two images from the positions indicated by white rectangles on the map are shown in (*e*) and (*g*). Their spot diagrams are shown in (*f*) and (*h*), respectively. The image in (*e*) is sampled from a multi-crystal diffraction zone (grey on the map), whereas the image in (*g*) is sampled from a region with single-crystal diffraction.

Densely packed crystals of thaumatin (from *Thaumatococcus daniellii*) were harvested in a micromesh, which was then subjected to a mesh scan (Fig. 9 ▸ *a*). Many of the diffraction images collected during the scan were polluted by intensive salt rings, reflection splitting, the superposition of diffraction patterns from many crystals and other artefacts of unclear origin (Figs. 9 ▸ *e* and 9 ▸ *g*). From the diffraction heat map itself (Fig. 9 ▸ *c*) it was unclear which regions would be suitable for diffraction data collection. In the spot lists produced by *Dozor* the strong reflections from salt or ice crystals were removed. *MeshBest* analysis revealed a steep slope of the baseline of the cumulative DDV histogram (Fig. 9 ▸ *b*), indicating that the large area in the central region of the sample holder (grey in Fig. 9 ▸ *d*) almost exclusively contained crystals stacked on each other. Nevertheless, 37 single crystals of varying dimensions were identified on the periphery of the mishmash, and are potentially sufficient for the collection of a complete data set.

## 4. Conclusion   

The results presented above clearly show that *MeshBest* analysis of X-ray mesh scans provides, in an automated way, useful information concerning the positions, sizes and relative diffraction strengths of crystals of macromolecules mounted in the sample holder. Such information is critical for the proper organization and design of subsequent diffraction data-collection protocols. Where *MeshBest* indicates that the sample holder contains one (or relatively few) crystal(s), the assessment of crystal size will allow a more precise description of radiation-damage effects arising during measurements (Zeldin *et al.*, 2013 ▸), especially when the size of the sample is smaller than X-ray beam, and thus help to define a more realistic data-collection strategy (Bourenkov & Popov, 2010 ▸). In cases where *MeshBest* indicates that large crystals are essentially homogeneous (*i.e.* §§[Sec sec3.1]3.1 and [Sec sec3.2]3.2), then such an approach combined with ‘helical’-style data collections (Flot *et al.*, 2010 ▸) might be the preferred mechanism of optimizing data quality. Here, though, the analysis presented in §[Sec sec3.2]3.2 shows that care should be exercised to define a protocol in which the direction of the helical scan and or the beam size used avoids illuminating satellite crystals and the production of multi-pattern diffraction images.

In cases where *MeshBest* indicates the presence of multiple small crystals packed tightly together on the sample support, data collection might be best carried out by accumulating many partial data sets from different individual crystals, with subsequent HCA-guided merging producing complete data sets (see, for example, Zander *et al.*, 2015 ▸). Here, in order to avoid noise from the diffraction patterns of adjacent crystals or from the medium in which the crystals are mounted, care should be taken to adjust the incident-beam size to the crystal size. Indeed, it can be imagined that future versions of *MeshBest* will pass information concerning crystal size directly to beamline-control interfaces in order to further help in the automation of the optimization of diffraction data collection in MX.

## Supplementary Material

The threshold for multi-pattern diffraction detection.. DOI: 10.1107/S2059798318002735/wa5115sup1.pdf

## Figures and Tables

**Table 1:** Mesh-scan experiment parameters for experiments aimed at testing the applicability of *MeshBest*

Case	1	2	3	4
Protein	Trypsin	Thermolysin	NarQ	Thaumatin
Crystal size (µm)	700 × 70 × 70	600 × 120 × 100	20–100 (range)	40–100 (range)
Space group	*P*2_1_2_1_2_1_	*P*6_1_22	*I*2_1_2_1_2_1_	*P*4_1_2_1_2
Unit-cell parameters (Å)	*a* = 62, *b* = 64, *c* = 69	*a* = *b* = 93, *c* = 130	*a* = 40, *b* = 59, *c* = 240	*a* = *b* = 58, *c* = 151
Beamline at ESRF	ID23-1	ID23-1	ID29	MASSIF-3
Wavelength (Å)	0.972	0.972	1.00	0.97
X-ray beam size (µm)	10 × 10	30 × 30	20 × 20	15 × 15
Mesh-scan grid dimensions (points)	13 × 22	13 × 21	26 × 19	33 × 43
Flux (photons s^−1^)	1.6 × 10^10^	5.6 × 10^10^	8.6 × 10^11^	4 × 10^11^
Sample rotation per image (°)	0.05	0.14	0.05	0.02
Detector edge resolution (Å)	1.6	1.5	2.5	1.7
Exposure time per image (ms)	37	37	62.4	50.6

**Table 2:** Data-collection and processing statistics for the production of a *MeshBest*-guided *MeshAndCollect* data set from crystals of the R50K mutant of the nitrate/nitrite-dependent histidine kinase NarQ from *E. coli* Values in parentheses are for the outer shell.

Diffraction source	ID29, ESRF
Wavelength (Å)	1.00
Detector	Dectris PILATUS 6M
Crystal-to-detector distance (mm)	378.8
Rotation range per image (°)	0.1
Total rotation range (°)	20
Exposure time per image (s)	0.05
No. of merged partial data sets	10
Space group	*I*2_1_2_1_2_1_
*a*, *b*, *c* (Å)	40.0, 59.7, 239.2
α, β, γ (°)	90, 90, 90
Resolution range (Å)	60.0–2.0 (2.1–2.0)
Total No. of reflections	138753 (19314)
No. of unique reflections	34226 (4734)
Completeness (%)	92.1 (93.4)
Multiplicity	4.05 (4.08)
〈*I*/σ(*I*)〉	7.37 (1.13)†
CC_1/2_ (%)	99.1 (73.2)
*R*_r.i.m._ (%)	8.4 (203.3)
Overall *B* factor from Wilson plot (Å^2^)	61.4

† The resolution cutoff was applied owing to the minimum resolution acquired at the edge of the detector.
