# Supplementary material for: The complex analysis of X-ray mesh scans for macromolecular crystallography
Source: Acta Crystallogr D Struct Biol. 2018 Apr 6;74(Pt 4):355–65. doi: 10.1107/S2059798318002735 (PMC6343787; doi:10.1107/S2059798318002735)
Supplement: Supplementary file 1 [file d-74-00355-sup1.pdf]

STRUCTURAL BIOLOGY

Volume 74 (2018)

Supporting information for article:

The complex analysis of X-ray mesh scans for macromolecular crystallography

Igor Melnikov, Olof Svensson, Gleb Bourenkov, Gordon Leonard and Alexander Popov

S1. The threshold for multi-pattern diffraction detection

The method of multi-pattern diffraction detection (see §2.1) employs the baseline fitting in the difference diffraction vector histogram (DDV histogram) of an image. The slope value of the linear fit of the baseline (hereinafter k_0, or the slope) is used as a marker for determining whether an image from a mesh scan (or any other single diffraction image) contains multi-pattern diffraction. In the case of superposition of diffraction patterns from two crystals we may assume that k_0 should be proportional to the number of spots in the weaker (or satellite, denoted as N_s) crystal pattern:

$$k_0 \propto N_s \qquad (A1)$$

To test the hypothesis (A1) simulations of diffraction images containing diffraction patterns from two separate crystals were constructed using pairs of diffraction images collected from single crystals (thermolysin, thaumatin and NarQ) during standard MX experiments.

Dozor was used to produce a list of detector spot coordinates and corresponding reflection intensities for each image. The spot lists of the second image in a pair were then computationally modified by applying an intensity filter (γ in Figure S1) and/or rotating the spots by the random angle around the X-ray beam axis. Such a manipulation allowed simulations of decreased diffraction strength and of randomness in satellite crystal orientation, respectively. The spot lists of both images in the pair were then merged to obtain simulated multi-pattern diffraction images. DDV histograms were then generated and the slope of the resulting baselines estimated (Figure S2). As can clearly be seen from Figure S2, the DDV histograms produced from the simulated multi-pattern diffraction images all have a baseline with a slope much greater than 0 and this baseline appears to be linear even in the case when the main and the satellite patterns have equal number of spots (Figure S2d).

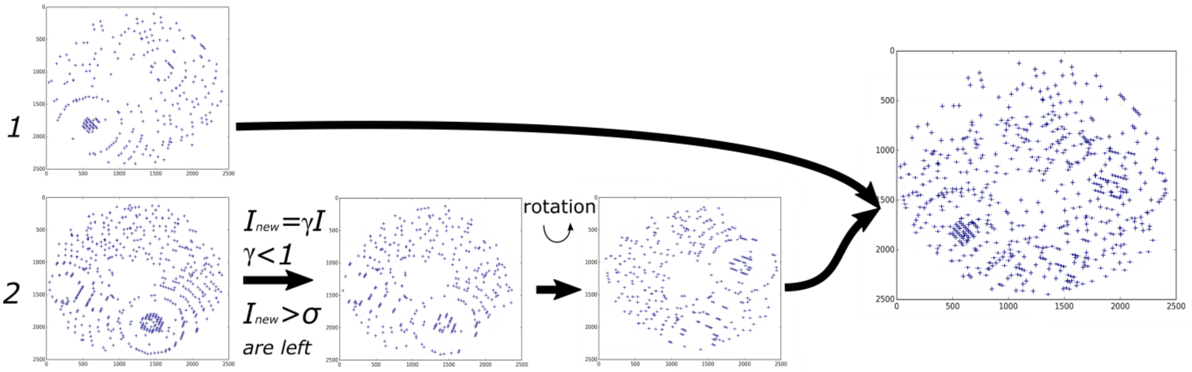

Figure S1 Simulating multi-crystal diffraction patterns. Two single-crystal patterns (left, 1 and 2) are chosen for combination. An intensity filter (γ < 1) is applied to pattern 2 and only spots of which the resulting intensity is still above background (σ, as determined by *Dozor*) are retained. Then, the new pattern 2 is rotated around the beam axis by the random angle. Finally, the lists of spot coordinates of patterns 1 and modified pattern 2 are merged to produce a simulated multi-crystal diffraction pattern (right).

The slope values were then normalised by the main crystal surface spot density $\rho = N_m/S$ where N_m is the number of spots in the main crystal pattern (pattern 1) and S the surface of the Ewald sphere cap containing all the detected spots, and plotted against the number of spots N_s in the satellite pattern (pattern 2) remaining after the intensity filter was applied (see Figure S3). As can be seen, the slope of the histogram baselines is clearly proportional to N_s, validating the correctness of the assumption (A1).

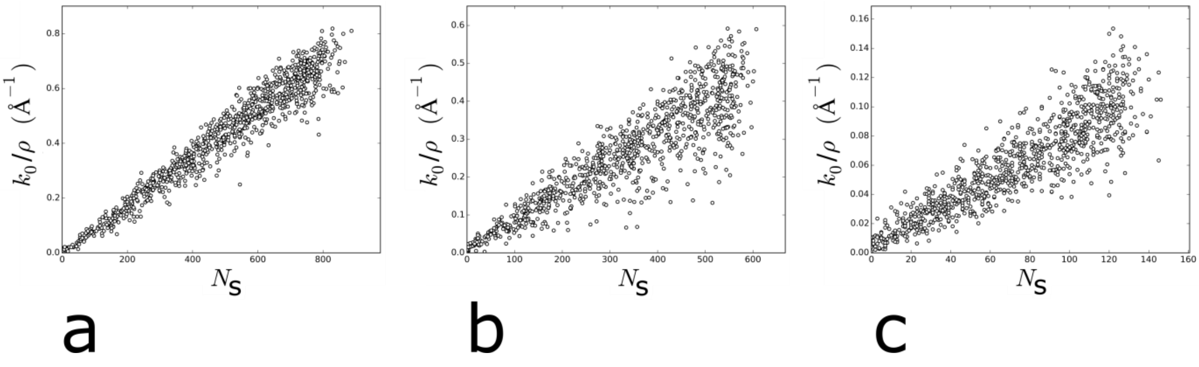

Figure S2 The sample DDV histograms calculated in the multi-pattern simulation experiment based on thermolysin (**a**), thaumatin (**b, d**) and NarQ (**c**) crystal diffraction patterns. The numbers N_m and N_s in the simulated pattern are presented. The case when the patterns have approximately equal number of spots is illustrated in **d**. The blue lines show the fitted linear approximations of the baselines.

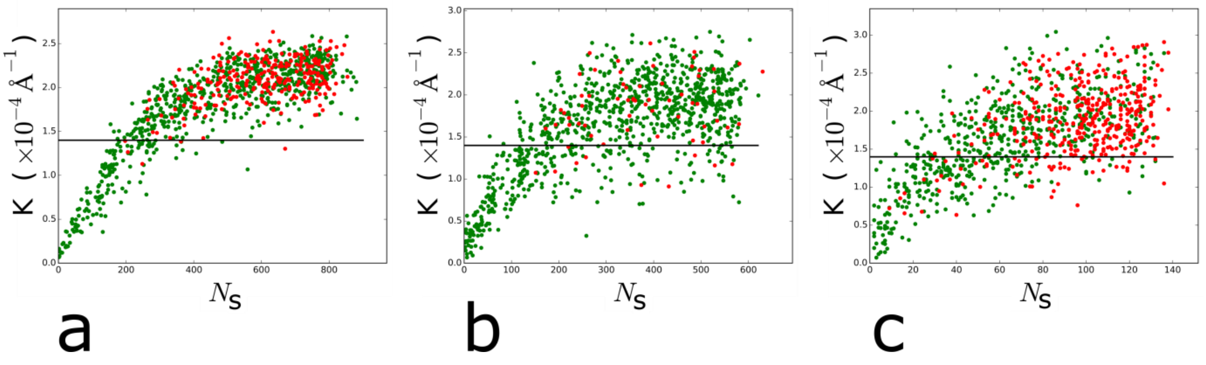

Figure S3 The dependence of the slope normalised by the spot surface density k_0/ρ (where $\rho = N_m/S$) from the number of satellite spots N_s in the simulations of multi-pattern diffraction. There are 1000 data points in total; each point represents one simulated pattern. The simulations were based on the data sets from standard rotational data collection from individual crystals of thermolysin (**a**), thaumatin (**b**) and NarQ (**c**).

The presented data (Figure S3) based on multi-pattern diffraction simulation supports the assumption of proportionality in (A1) and further states taking into account the normalisation by spot density ρ:

$$\frac{k_0}{\rho} \propto N_s \Rightarrow k_0 \propto \rho N_s = \frac{N_m N_s}{S} \qquad (A1a)$$

Unfortunately, however, using k_0 presented as a determinant of multi-pattern diffraction is not ideal because the exact numbers of spots from each lattice contributing to the diffraction image is usually unknown and only the total number N of spots in the image is available. To account for all this a better measure is

$$\mathrm{K} = \frac{k_0}{\rho N} = k_0 \cdot \frac{S}{N^2} \qquad (A2)$$

which is k_0 normalised by spot density N/S and the total number of spots, N, on a diffraction image. Taking into account, that from (A1a):

$$k_0 = \alpha \frac{N_m N_s}{S}$$

where α is the proportionality constant, and that in two-crystal case:

$$N = N_m + N_s$$

one can state from (A2):

$$\mathrm{K} = \alpha \frac{N_m N_s}{S} \cdot \frac{S}{(N_m + N_s)^2} = \alpha \frac{N_m N_s}{(N_m + N_s)^2} = \alpha \frac{{}^{N_s}/_{N_m}}{\left(1 + {}^{N_s}/_{N_m}\right)^2} \qquad (A3)$$

In equation (A3) the presumable dependence of K on the fraction of satellite crystal spots N_s/N_m is shown. This hypothetical behaviour of K was analysed by multi-pattern diffraction simulations. The patterns were again simulated in the manner described above, and the value of K was calculated based on the histogram baseline fit. The results of this analysis are shown in Figure S4 in which the dependence of K on N_s is in accordance with (A3) taking into account that the number of spots in the main pattern N_m was nearly constant for each protein.

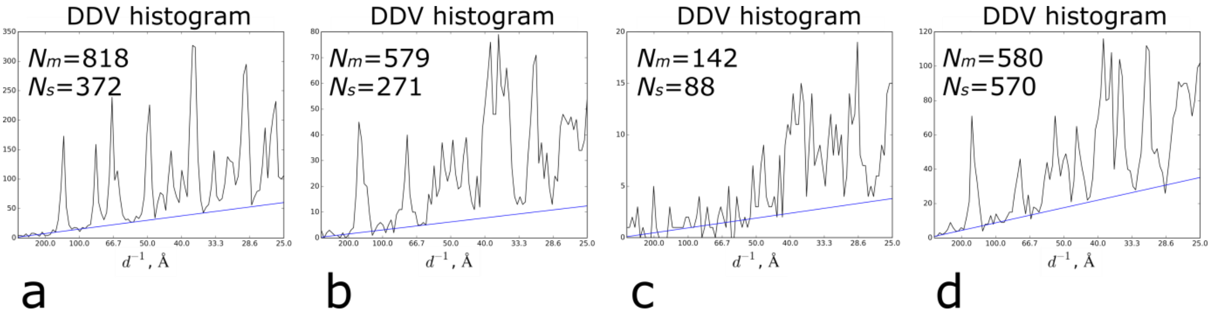

The dependence of the K value and of the possibility of pattern indexing from the number of satellite spots N_s in the simulations of multi-pattern diffraction. Each point represents one simulated pattern, 1000 points in total. The successful indexing of the simulated pattern is shown by green points, the failed indexing is shown by red points. The simulations were based on the data sets from standard rotational data collection from individual crystals of thermolysin (**a**), thaumatin (**b**) and NarQ (**c**) which had nearly 900, 600 and 150 as the number N_m of spots in the original diffraction pattern, respectively, as determined by *Dozor*. The black horizontal line indicates the threshold level.

The relation between the K value, the number of spots N_s in the satellite pattern and the possibility to index multi-pattern diffraction images was examined using *XDS* (Kabsch, 2010). Here, simulated multi-pattern diffraction images were introduced by modification of the spot-list file SPOT.XDS and a total of 5 multi-pattern images (which corresponded to maximum 0.5° of rotation) was used for indexing starting with the image on which the histogram (see Figure S2) was calculated. The results are also presented in Figure S4 which shows, at least for the three crystalline systems on which the simulations were based, that *XDS* successfully indexes multi-pattern diffraction images producing difference vector histograms with $K < 1.4 \cdot 10^{-4}$ Å^{-1} and this threshold was set for determining at which point multi-pattern diffraction regions of mesh scans should be removed from subsequent data collection protocols. The threshold chosen corresponds (Figure S4) to a two-pattern diffraction image in which the fraction of satellite crystal spots $N_s/N_m = 0.3$.
